# Identifications of novel mechanisms in breast cancer cells involving duct-like multicellular spheroid formation after exposure to the Random Positioning Machine

**DOI:** 10.1038/srep26887

**Published:** 2016-05-27

**Authors:** Sascha Kopp, Lasse Slumstrup, Thomas J. Corydon, Jayashree Sahana, Ganna Aleshcheva, Tawhidul Islam, Nils E. Magnusson, Markus Wehland, Johann Bauer, Manfred Infanger, Daniela Grimm

**Affiliations:** 1 Clinic for Plastic, Aesthetic and Hand Surgery, Otto-von Guericke-University, D-39120 Magdeburg, Germany; 2 Department of Biomedicine, Aarhus University, DK-8000 Aarhus C, Denmark; 3 Medical Research Laboratory, Department of Clinical Medicine, Aarhus University, DK-8000 Aarhus C, Denmark; 4 Max-Planck-Institute of Biochemistry, D-82152 Martinsried, Germany

## Abstract

Many cell types form three-dimensional aggregates (MCS; multicellular spheroids), when they are cultured under microgravity. MCS often resemble the organ, from which the cells have been derived. In this study we investigated human MCF-7 breast cancer cells after a 2 h-, 4 h-, 16 h-, 24 h- and 5d-exposure to a Random Positioning Machine (RPM) simulating microgravity. At 24 h few small compact MCS were detectable, whereas after 5d many MCS were floating in the supernatant above the cells, remaining adherently (AD). The MCS resembled the ducts formed *in vivo* by human epithelial breast cells. In order to clarify the underlying mechanisms, we harvested MCS and AD cells separately from each RPM-culture and measured the expression of 29 selected genes with a known involvement in MCS formation. qPCR analyses indicated that cytoskeletal genes were unaltered in short-term samples. *IL8, VEGFA*, and *FLT1* were upregulated in 2 h/4 h AD-cultures. The *ACTB, TUBB, EZR, RDX, FN1, VEGFA, FLK1 Casp9, Casp3, PRKCA* mRNAs were downregulated in 5d-MCS-samples. *ESR1* was upregulated in AD, and *PGR1* in both phenotypes after 5d. A pathway analysis revealed that the corresponding gene products are involved in organization and regulation of the cell shape, in cell tip formation and membrane to membrane docking.

Breast cancer is the second most common cancer worldwide with 1.7 million cases in 2012^1^. Advances in prevention, early diagnosis, surgical treatment and postsurgical therapies enhanced the possibility of a complete cure^2^. Known molecular targets (e.g. VEGF, VEGFR, HER2/neu) for approved drugs (e.g. tyrosine kinase inhibitors like sorafenib), or approved therapeutic antibodies (e.g. bevacizumab, ramucirumab, trastuzumab) are proteins, which are predominantly expressed in breast cancer cells and are simultaneously involved in promoting cell growth or apoptosis^3^,^4^. However, it is difficult at the current state of technology to apply the optimal cocktail of drugs to hit all cancer cells of any given patient. Under these circumstances, it is absolutely necessary to find new proteins, which can serve as targets to develop drugs against this cancer type.

In earlier studies we proved repeatedly that exposing various cell types like thyroid cells, endothelial cells and chondrocytes to simulated microgravity (s-μ*g*) results in a scaffold-free production of three-dimensional (3D) aggregates so-called multicellular spheroids (MCS)^5^,^6^,^7^,^8^,^9^,^10^. The MCS very often resemble the tissue, from which the cells have been derived. In case of cancer cells, the *in vivo* structure of tumors appears more closely represented by MCS than by monolayer cell cultures^11^,^12^,^13^. A proteomics investigation on thyroid cancer cells had shown that FTC-133 cells express surface proteins binding fibronectin which induces 3D cohesion^5^.

Vassy and coworkers were the first scientists who investigated MCF-7 human breast cancer cells exposed to microgravity. When these cells came back from a Photon capsule mission, their cytoskeleton was changed^14^. Later Qian *et al*.^15^ demonstrated that culturing MCF-7 cells on a clinostat affected several cell features including cancer cell migration and adhesion^15^. Moreover, Li *et al*. found that MCF-7 cells are sensitive to simulated microgravity in regard to integrin expression and microtubule formation^16^. Furthermore, Zheng *et al*. reported a protective role of the estrogen receptor on MCF-7 cells exposed to simulated microgravity^17^.

Masiello *et al*. demonstrated 3D aggregates and adherently growing MDA-MB-231 breast cancer cells after a 24 h- and 72 h-RPM-exposure^18^. These morhological differences were accompanied by changes in biological processes such as proliferation and apoptosis as well as signaling pathways^18^.

In this study, we used the method of annulling gravity by a Random Positioning Machine (RPM) to find alterations of the MCF-7 breast cancer cell growth behavior in concert with changes in the expression of selected genes, playing a role in angiogenesis and tumor metastasis^7^, because the RPM not only prevents cell sedimentation, but also ensures a favorable environment for cell cultures, as the movements of the platforms enable sufficient oxygen, nutrient and waste transport^19^,^20^. We cultured the MCF-7 cell line on the RPM for 2 h, 4 h, 16 h, 24 h, and 5d respectively to focus on short-term and long-term effects of simulated microgravity on breast cancer cells. The cell line was derived from a pleural effusion of a patient with metastatic mammary carcinoma. It is described to build up 3D-dome structures upon absolute confluence, which however remain attached to the bottom. In addition, the cells retained breast cell common features like estrogen receptor and progesterone receptor^21^.

After exposing the MCF-7 breast cancer cells to the RPM, cells which remained adherently to the bottom of the culture dish (AD) and cells included in 3D aggregates were harvested separately. This different growth behavior was also found in endothelial cells and thyroid cells^6^,^7^,^13^,^22^. Morphology and gene expression patterns of AD and MCS cells were analyzed in comparison to each other and to cells grown in a normal laboratory incubator as 1 *g* (gravity)-controls. The principal aim of this study was to identify the underlying mechanisms of spheroid formation, when human breast cancer cells were cultured under conditions of simulated microgravity on the RPM. Using pathway analysis programs the interactions of genes and proteins were studied in detail.

## Results

### MCF-7 tumor cells form 3D aggregates by RPM-exposure

#### Short-term study

**Figure 1:**
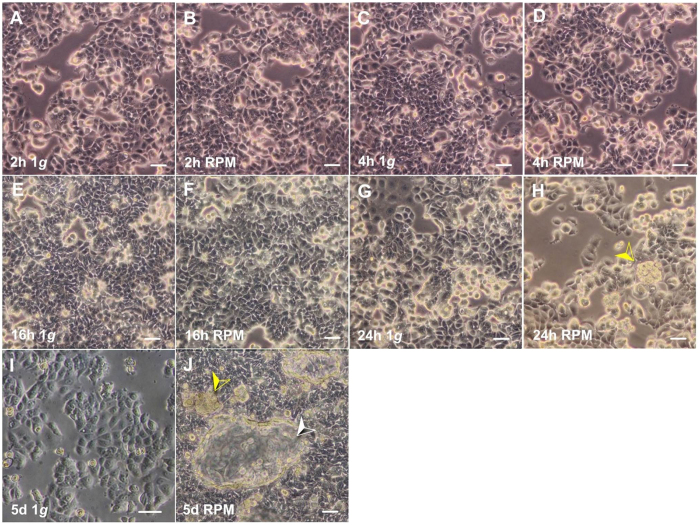
Morphologic examination of the cells. Phase-contrast microscopy of MCF-7 breast cancer cells cultured under normal static 1 *g*-conditions for 2 h (**A**), 4 h (**C**), 16 h (**E**), 24 h (**G**), 5d (**I**) and on the RPM for 2 h (**B**), 4 h (**D**), 16 h (**F**), 24 h (**H**) and 5d (**J**). Control samples of 5d (**I**) formed no MCS. Samples cultured for 5d on the RPM (**J**) revealed cells that stayed adherently as a monolayer, and solid MCS (yellow arrow) as well as hollow MCS (white arrow). Scale bar: 50 μm.

Phase contrast microscopy revealed epithelial-like MCF-7 cells growing in monolayers under normal static 1 *g*-conditions (Fig. 1A,C,E,G). MCF-7 cells are small and have a polygonal shape. MCF-7 cells exposed to the RPM for 2 h, 4 h, and 16 h showed no three-dimensional growth and only an adherent phenotype (Fig. 1B,D,F), whereas after a 24 h-RPM-exposure small compact round three-dimensional (3D) multicellular spheroids (MCS) were found floating in the supernatant (Fig. 1H). Two phenotypes were now detectable – adherently growing MCF-7 cells (AD) and 3D MCS.

#### Long-term study

After culturing MCF-7 cells on the RPM for 5 days (d) respectively, the cellular morphology of the 1 *g*-cultures was not altered (Fig. 1I). After culturing MCF-7 cells for 5d on the RPM, two distinct cell morphologies were clearly detectable. One AD cell population and another 3D growing population which had detached from the bottom and built solid (Fig. 1J, yellow arrow) and hollow, loose (Fig. 1J, white arrow) 3D MCS.

**Figure 2:**
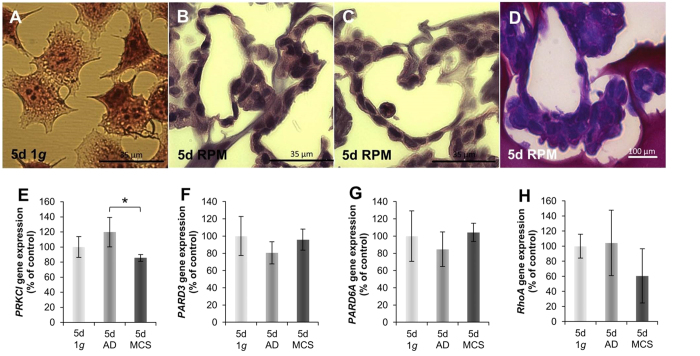
Structural investigations of the MCS. (**A**–**C**) HE staining: (**A**) 5d, 1 *g*-control cells; (**B**,**C**) examples of 3D MCS with glandular structures. Scale bar: 35 μm (**D**) PAS-stained MCS with apical-basal polarity of the cancer cells. Scale bar 100 μm (**E**) *PRKCI* gene-expression; (**F**) *PARD3* gene-expression; (**G**) *PARD6A* gene expression and (**H**) *RhoA* gene expression. *p < 0.05.

These 3D aggregates were further investigated by histochemistry using hematoxylin-eosin (HE) and Periodic Acid-Schiff (PAS) staining. Figure 2A shows normal HE-stained MCF-7 breast cancer cells. Figure 2B–D show the typical glandular structure of MCS with a clear lumen. The breast cancer cells reveal an apical-basal cell polarity. Whereas mechanisms of cell polarity are quite complex, the Par3(Bazooka)-Par6-aPKC protein complex plays an important role in the establishment and maintenance of apical-basal cell polarity^23^. The Par3(Bazooka)-Par6-aPKC protein complex localizes to the apical membrane domain and promotes the apical-membrane-domain identity. Here we determined the gene expression of the players of the complex and found a down-regulation of *PRKCI* mRNA in 5d-MCS-samples compared to AD and 1 *g*-samples (Fig. 2E). The *PARD3, PARD6A* and *RhoA* mRNAs were not significantly changed (Fig. 2F–H).

### Changes of the cytoskeleton and associated proteins

**Figure 3:**
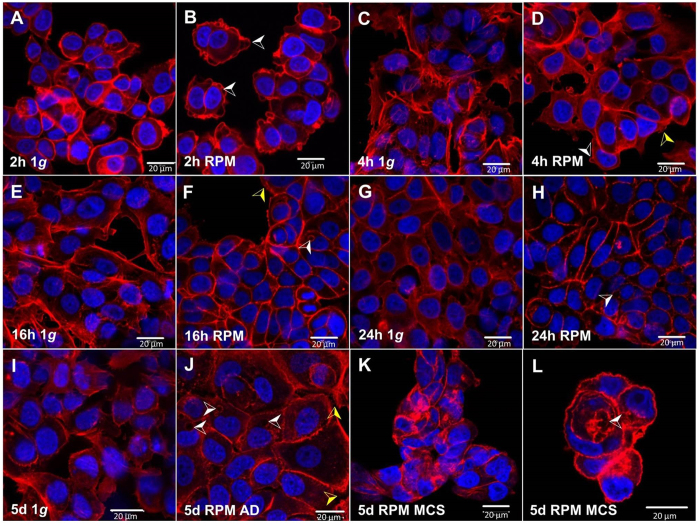
F-actin cytoskeleton. Confocal laser scanning microscopy of rhodamine-phalloidin stained MCF-7 cells after a 2 h-, 4 h-, 16 h-, 24 h- and 5d-RPM-exposure and corresponding 1 *g*-control cells. (**A**) 2 h 1 *g*; (**B**) 2 h RPM-culture, white arrow indicated membrane blebbing; (**C**) 4 h 1 *g*; (**D**) 4 h RPM-culture, the white arrow indicated membrane bleebing, the yellow arrow shows stress fibers; (**E**) 16 h 1 *g*; (**F**) 16 h RPM-culture, the yellow arrow shows stress fibers, the white arrow presents cytoskeletal holes; (**G**) 24 h 1 *g*; (**H**) 24 h RPM-culture, the white arrow indicates cytoskeletal holes; (**I**) 5d, 1 g; (**J**) 5d RPM AD cells, the white arrow indicates holes, the yellow arrows show stress fibers; (**K**,**L**) 5 d RPM MCS, the white arrow indicates a glandular structure. Scale bar: 20 μm; blue staining: DAPI highlights the nucleus; red staining: rhodamine-phalloidin to visualize the F-actin.

In order to detect further changes of the cell shape and the cytoskeleton, the cells had been fixed and stained for F-actin (visualized by means of rhodamine-phalloidin staining) and 4′,6-diamidino-2-phenylindole (DAPI) staining after cultivation for 2 h, 4 h, 16 h and 24 h as well as for 5d on the RPM or under static 1 *g*-conditions (Fig. 3).

#### Short-term study

The cells appeared to be more evenly distributed under conditions of 1 *g* than after RPM-exposure. The cell membrane structure was changed after a 2 h-RPM-exposure (Fig. 3B). A membrane blebbing (white arrows) was detectable in 2 h-RPM-samples, whereas no blebbing was found in corresponding static 1 *g*-controls (Fig. 3A). Stress fibers were detectable after 4 h (yellow arrow) in the cell periphery in cells exposed to the RPM in concert with a decreased membrane blebbing, but no changes were visible in control cells. The stress fibers decreased with the duration of the experiment and were less prominent 16 h and 24 h. However, the bundles of actin filaments were thin and did not show a long-range orientation. After 24 h culturing on the RPM, cytoskeletal holes were visible (Fig. 3H, white arrow).

#### Long-term study

5d 1 *g*-control cells showed a normal microfilament system with visible actin fibers, evenly distributed in the cells (Fig. 3I). In contrast, RPM-exposed adherent cells presented an accumulation of F-actin at the cell boundaries (Fig. 3J). Some cells displayed pronounced holes (Fig. 3J, white arrow) and stress fibers (Fig. 3J, yellow arrow), while their nuclei were intact. The MCS after 5d-exposure revealed solid aggregates of living cells with an accumulation of F-actin towards the cell boundaries, but no distinct polymerization direction (Fig. 3K) and MCS with a small lumen (Fig. 3, white arrow L).

### Investigation of the underlying mechanisms of the phenotypical changes of the cells

**Figure 4:**
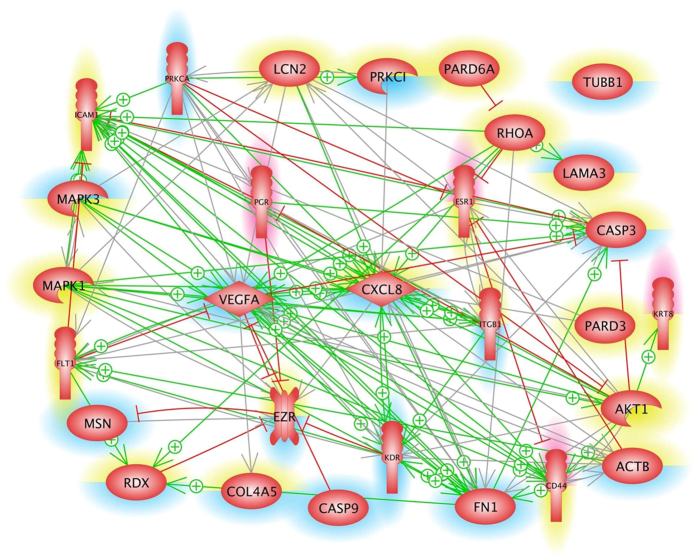
Mutual interaction of selected genes at gene expression level. 29 selected genes, whose up- or downregulation were analysed by qRT-PCR after 5d of culturing on the RPM and shown in Figs 2 and 6, 7, 8, 9. Blue background indicates down-regulation, red background shows up-regulation. The yellow background refers to non-regulated genes. The lower part of each icon indicates the gene status in MCS cells, whereas the upper part indicates the status of the gene in the AD cells. The green arrows indicate activating and the red one inhibiting effects. The interaction network was built up using Elsevier Pathway Studio v.11.

**Figure 5:**
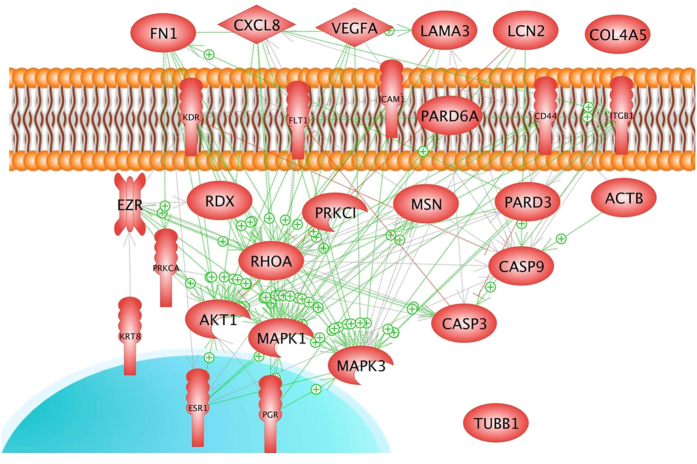
Mutual interaction and localization of proteins coded by the 29 selected genes. The green arrows indicate activating and the red one inhibiting effects. The interaction network was built up using Elsevier Pathway Studio v.11.

**Figure 6:**
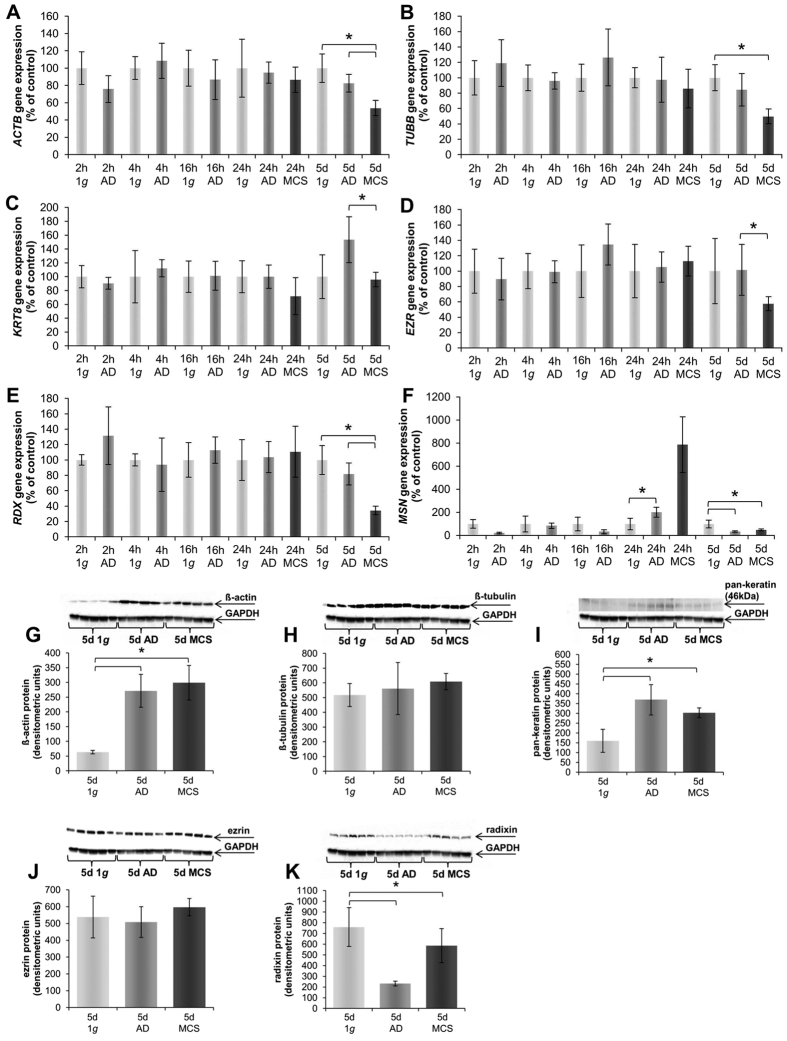
Quantitative alterations of gene expression and protein content of cytoskeletal and associated proteins: Genes. *ACTB* (**A**) 2 h, 4 h, 16 h, 24 h and 5 d RPM-experiments. *TUBB* (**B**) 2 h, 4 h, 16 h, 24 h and 5d RPM-experiments. *KRT8* (**C**) 2 h, 4 h, 16 h, 24 h and 5d RPM- experiments. *EZR* (**D**) 2 h, 4 h, 16 h, 24 h and 5d RPM-experiments. *RDX* (**E**) 2 h, 4 h, 16 h, 24 h and 5d RPM-experiments. *MSN* (**F**) 2 h, 4 h, 16 h, 24 h and 5d RPM-experiments. **Proteins of 5d-experiments:** 5d β-actin (**G**); 5d β-tubulin (**H**); 5d cytokeratin (**I**); 5d Ezrin (**J**) 5d Radixin (**K**); *p < 0.05.

**Figure 7:**
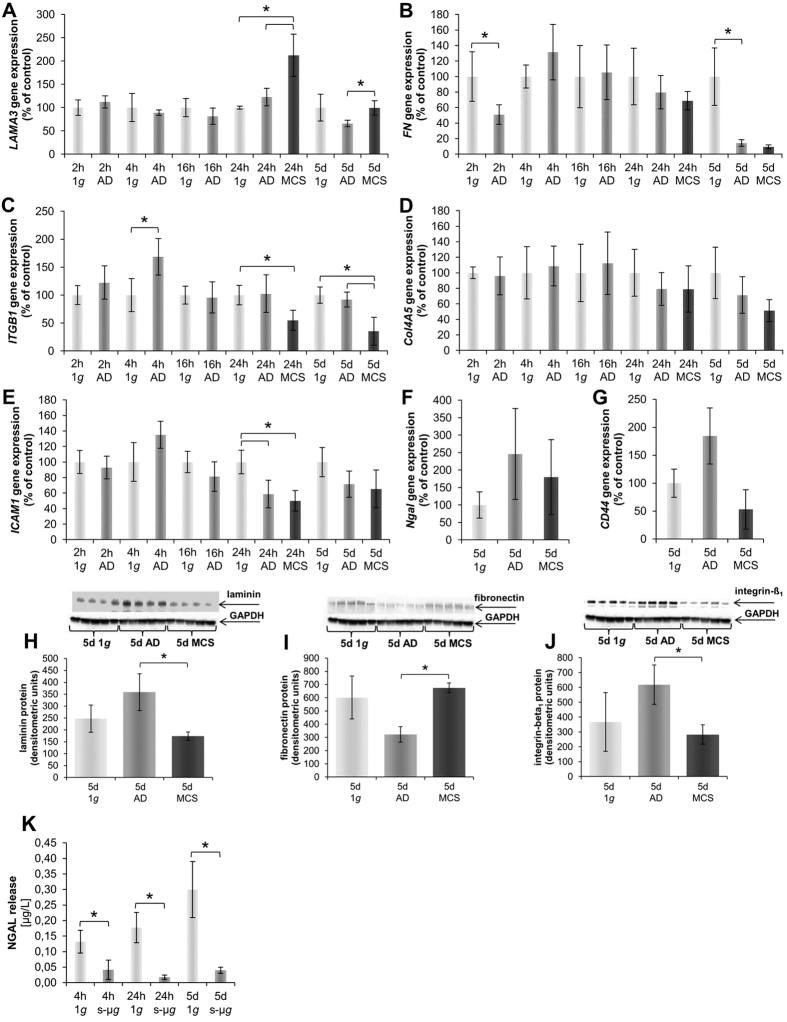
Quantitative alterations of gene expression and protein content of extracellular matrix and associated proteins: Genes. *LAMA3* (**A**) 2 h, 4 h, 16 h, 24 h and 5d RPM-experiments. *FN1* (**B**) 2 h, 4 h, 16 h, 24 h and 5d RPM-experiments. *ITGB1* (**C**) 2 h, 4 h, 16 h, 24 h and 5d RPM-experiments. *Col4A5* (**D**) 2 h, 4 h, 16 h, 24 h and 5d RPM-experiments. *ICAM1* (**E**) 2 h, 4 h, 16 h, 24 h and 5d RPM-experiments. *Ngal* (**F**) 5d RPM-experiment. *CD44* (**G**) 5d RPM-experiment. **Proteins:** 5d laminin (**H**); 5d fibronectin (**I**); 5d integrin-β-1 (**J**). NGAL release (**K**) 4 h, 24 h and 5d. *p < 0.05.

**Figure 8:**
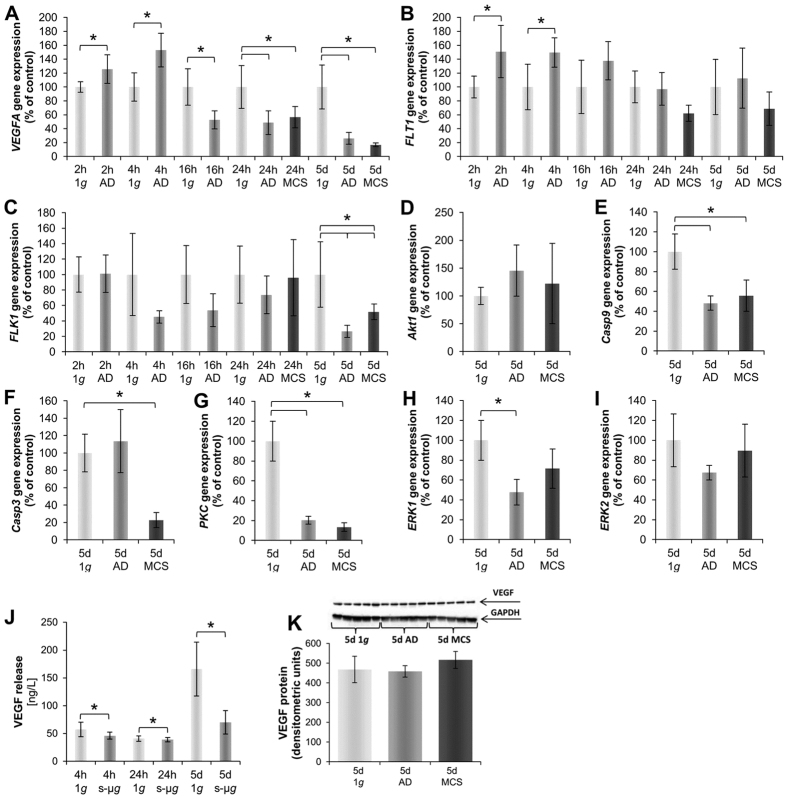
Quantitative alterations of gene expression and protein content of VEGF signalling pathway molecules: Genes. *VEGFA* (**A**) 2 h, 4 h, 16 h, 24 h and 5d RPM-experiments. *FLT1* (**B**) 2 h, 4 h, 16 h, 24 h and 5d RPM-experiments. *FLK1* (**C**) 2 h, 4 h, 16 h, 24 h and 5d RPM- experiments. *Akt1* (**D**) 5d. *Casp9* (**E**) 5d. *Casp3* (**F**) 5d. *PKC* (**G**) 5d. *ERK1* (**H**) 5d. *ERK2* (**I**) 5d. **Proteins:** VEGF release in the supernatant (**J**) 4 h, 24 h and 5d; 5d VEGF protein content (**K**). *p < 0.05.

**Figure 9:**
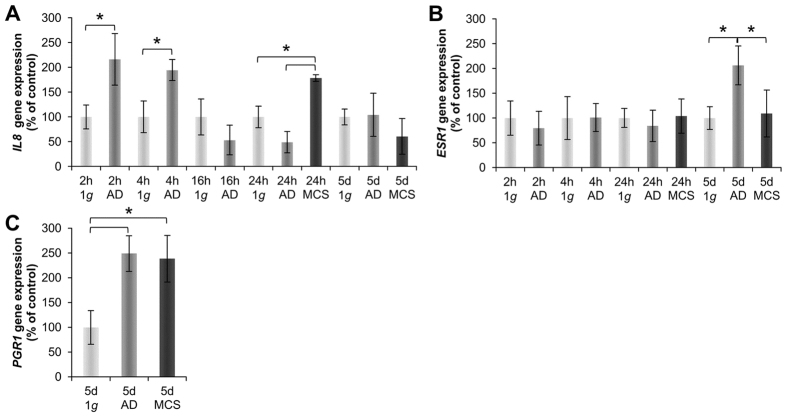
Quantitative alterations of gene expression of cytokines and hormone receptors: Genes. *IL8* (**A**) 2 h, 4 h, 16 h, 24 h and 5d RPM-experiments. *ESR1* (**B**) 2 h, 4 h, 16 h, 24 h and 5d RPM-experiments. *PGR1* (**C**) 5d RPM-experiments. *p < 0.05.

In order to find the mechanisms for the transition of the cells from a 2D to a 3D kind of growth behavior, we selected 29 genes (Table 1), which code for proteins known to be involved either in regulation and maintaining cell structures and shapes or in cell migration or in apoptosis^5^,^6^,^7^,^8^,^9^,^10^,^24^ or were specific for female epithelial cells^17^,^21^. A pathway analysis revealed that aside from β-tubulin (*TUBB*), the expression of the other 28 genes is mutually controlled within the frame of a network (Fig. 4). The proteins coded by these genes consisted of 6 extracellular proteins, 6 membrane proteins, 15 cytoplasmic proteins and 2 nuclear proteins. They also form a network of regulation which stretches from the outside, across the membranes towards the nucleus (Fig. 5). In order to see which influence an up- or down-regulation of a given gene could have on the rest of the network, we analysed the interaction of the selected genes and determined how their up- and down-regulation is linked. Figure 4 gives an overview on the status of regulation of the 29 genes determined by the PCR after 5d of culturing on the RPM and shown in Figs 2 and 6, 7, 8, 9. Blue background indicates down-regulation, red background shows up-regulation. The yellow background refers to non-regulated genes. The lower part of each icon indicates the gene status in MCS cells, whereas the upper part indicates the status of the gene in the AD cells. The green arrows indicate activating and the red one inhibiting effects. The picture clearly indicates that the cytokine interleukin-8 (*IL-8 or* CXCL8) gene influences the most of the neighboring genes and thus, may play a central role within this complicated network of regulation. It is followed by *FN1, VEGFA, ICAM1* and *Casp3* genes as we have seen in earlier studies on cells exposed to the RPM^13^. Of these genes *IL-8* and *Casp3* were only downregulated in MCS, whereas *VEGFA* and *FN1* mRNAs were reduced in both populations.

### Simulated microgravity on the RPM changes the gene expression and protein production of cytoskeletal and of cytoskeleton-binding proteins

#### Short-term study

Genes associated with the cytoskeleton such as β-actin (*ACTB*), β-tubulin (*TUBB*), cytokeratin-8 (*KRT8*), ezrin (*EZR*), and radixin (*RDX*) mRNAs were not significantly changed after short-term incubation (2 h, 4 h, 16 h and 24 h) on the RPM (Fig. 6A–E). The moesin (*MSN*) mRNA was reduced at early time points, but was up-regulated after 24 h (Fig. 6F).

#### Long-term study

As compared to 1 *g*-control cells, the gene expression of *ACTB* after a 5d-cultivation on the RPM revealed no changes in AD cells, while a significant down-regulation in MCS was measured compared to AD and 1 *g*-samples (Fig. 6A). Taking a look at the corresponding proteins by Western blot analysis revealed that the β-actin protein content was increased in both phenotypes of 5d-RPM-samples compared to 1 *g*-controls (Fig. 6G).

The *TUBB* gene expression presented a comparable picture to β-actin. After 5d no changes were visible for AD cells. However, MCS after 5d showed a significant down-regulation compared to 1 *g*-controls (Fig. 6B). Western blot analyses revealed no changes in the protein content of RPM-exposed samples compared to their corresponding controls (Fig. 6H).

The *KRT8* gene expression was slightly elevated in 5d-AD-samples, but significantly down-regulated in MCS compared to AD samples and not significantly changed compared to 1 *g*-controls (Fig. 6C). In addition, the amount of pan-cytokeratin protein was enhanced after a 5d- culture on the RPM in both phenotypes in comparison to 1 *g*-controls (Fig. 6I). The *EZR* gene expression of AD samples was not significantly changed compared to the 1 *g*-control group (Fig. 6D), but MCS samples exhibited a decrease in *EZR* mRNA. No change was found in the protein after 5d, respectively (Fig. 6J). The *RDX* gene expression in MCS after 5d was significantly down-regulated (Fig. 6E). In addition, the protein content was decreased in AD cells after a 5d-exposure of MCF-7 cells on the RPM as compared to MCS and 1 *g*-controls (Fig. 6K). The *MSN* gene expression after 5d was significantly down-regulated in AD and MCS cells compared to the 1 *g*-controls (Fig. 6F).

### Cultivation of MCF-7 cells on the RPM induces changes in the extracellular matrix

#### Short-term study

In this study we investigated the expression of extracellular matrix (ECM) proteins. The laminin α3 (*LAMA3*) gene expression was unaltered in cells exposed for 2 h, 4 h and 16 h to the RPM. In addition, the *LAMA3* mRNA was significantly elevated in MCS compared to 1 *g-* and AD-samples after 24 h (Fig. 7A). The fibronectin (*FN1*) mRNA is decreased after a 2 h-RPM-exposure compared to 1 *g*-samples, but remained unalterd at the other short-term time points (Fig. 7B).

Levels of integrin-β_1_ (*IGTB1*) transcripts of AD cells were slightly elevated compared to 1 *g*-samples after 2 h, then significantly up-regulated after 4 h, and then unaltered after a 16 h-RPM-exposure (Fig. 7C). After 24 h, the *ITGB1* mRNA was down-regulated in MCS compared to AD and 1 *g* (Fig. 7C).

The ECM protein collagen type 4 (*COL4A5*) mRNA was not significantly altered under all conditions (Fig. 7D). The gene expression of intercellular adhesion molecule 1 (*ICAM1*) was significantly down-regulated in both RPM-cultures after a 24 h-RPM exposure (Fig. 7E).

The neutrophil gelatinase-associated lipocalin (NGAL) secretion was significantly decreased after 4 h and 24 h of incubation on the RPM compared to 1 *g* (Fig. 7K). The release of NGAL was below the detection level of the technique after 2 h of incubation of the MCF-7 cells on the RPM.

#### Long-term study

The *LAMA3* mRNA was decreased in AD compared to MCS and 1 *g*-samples after a 5d-RPM-exposure (Fig. 7A). The amount of laminin protein was elevated in AD cells and significantly reduced to the 1 *g*-level in MCS (Fig. 7H). The *FN1* expression was significantly down-regulated after a 5d-RPM-exposure in AD cells and in MCS (Fig. 7B). In contrast, the amount of fibronectin protein was decreased in AD cells after 5d. However, MCS showed an normalization of the protein synthesis like 1 *g*-samples (Fig. 7I).

Concerning the gene expression of *ITGB1*, there was a slight down-regulation visible after 5d and a further decrease in MCS cells compared to 1 *g*-samples (Fig. 7C). Determination of the protein content showed that MCS cells exhibited a significant decrease in β_1_-integrin protein compared to AD samples (Fig. 7J). The gene expression of the extracellular matrix protein *Col4A5* was not significantly changed after a 5d-RPM exposure (Fig. 7D). In addition, the *ICAM1* gene expression was not altered after 5d (Fig. 7E). Moreover, the *Ngal* gene expression was not remarkably changed after a 5d-RPM-exposure (Fig. 7F). In addition, the release of NGAL protein into the supernatant was significantly decreased in RPM-exposed samples compared to the 1 *g*-controls after 5d (Fig. 7K). Furthermore, *CD44* was not significantly changed after 5 days. AD cells presented a tendency to elevate the expression (Fig. 7G).

### Vascular endothelial growth factor signalling pathway molecules are altered by simulated microgravity

#### Short-term study

Both genes, vascular endothelial growth factor A (*VEGFA*) and its receptor vascular endothelial growth factor receptor 1 or fms related tyrosine kinase 1 (*FLT1*) were significantly up-regulated after a 2 h-incubation on the RPM (Fig. 8A,B), while the vascular endothelial growth factor receptor 2 or fetal liver kinase 1 (*FLK1*) mRNA was unchanged at all short-term time points (Fig. 8C). The *VEGFA* and *FLT1* mRNAs were still significantly up-regulated after a 4 h-RPM-exposure. Interestingly later after 16 h we detected a down-regulated *VEGFA* mRNA in AD samples and after 24 h in both AD and MCS samples (Fig. 8A).

The amount of secreted VEGF protein was measured in the cell culture supernatants of 1 *g*- and RPM-experiments by time resolved immunofluorometric assays (TRIFMA). The results showed that the amount of VEGF protein was significantly decreased in RPM-cultures compared to 1 *g*-controls after a 4 h-RPM-exposure (Fig. 8J). There was no difference between 1 *g*- and RPM-cultures after 24 h. However, comparing the VEGF levels in s-μ*g* after 4 h and 24 h against each other, a significant lower level after 24 h could be observed.

#### Long-term study

The gene expression of *VEGFA* was significantly down-regulated after 5d of culturing MCF-7 cells on the RPM (Fig. 8A). This is in concert with the VEGF protein release in the supernatant as measured by TRIFMA. The release of VEGF protein was significantly lower in RPM-samples compared to corresponding static 1 *g*-controls (Fig. 8J). The Western blot analysis revealed no significant change in AD cells, but a slight elevation of VEGF protein in MCS compared to 1 *g*-cultures (Fig. 8K).

The gene *FLK1* was significantly down-regulated after 5d in both cell populations (Fig. 8C). In contrast to *FLK1*, the *FLT1* mRNA was not altered in cells cultured on the RPM at this time point (Fig. 8B).

The threonine-protein kinase (*akt1* or *PKB*) gene coding for a signaling cascade molecule was unaltered after 5d of cultivation on the RPM (Fig. 8D).

The caspase-9 (*Casp9*) gene expression was significantly down-regulated in MCF-7 cells after RPM-exposure compared to 1 *g*-controls (Fig. 8E). In contrast to caspase-9, the caspase-3 (*Casp3*) gene expression was significantly down-regulated only in MCS after 5d (Fig. 8F). The gene expression of protein kinase C (*PKC* or *PRKCA*) was significantly down-regulated in both cell populations after 5d compared to their corresponding 1 *g*-controls (Fig. 8G).

The extracellular-signal regulated kinase (*Erk*)-1 (Fig. 8H) and *Erk-2* (Fig. 8I) presented a similar behavior after RPM-exposure. After 5d only the AD cell populations exhibited a significantly down-regulated expression of *Erk-1*, the other groups showed a slight decrease compared to 1 *g*-controls (Fig. 8H,I).

### RPM-exposure induces expression changes of interleukin-8, estrogen- and progesterone receptors

The cytokine interleukin-8 (*IL8*) gene expression was not altered in AD, but slightly decreased in MCS cells on the RPM after 5d (Fig. 9A). After 2 h and 4 h a clear up-regulation of the *IL8* gene was detectable in AD RPM-cultures. After 16 h this elevation was attenuated again to the normal 1 *g*-level. In 24 h-samples MCS exhibited an up-regulated *IL8* mRNA compared to AD and 1 g-samples (Fig. 9A).

The estrogen receptor (*ESR1*) expression was unchanged in all short-term samples, but was up-regulated after 5 days in AD cells. In MCS the *ESR1* mRNA was unaltered (Fig. 9B). The progesterone receptor (*PGR1*) expression was significantly up-regulated in both AD cells as well as in MCS cells after 5 days (Fig. 9C).

## Discussion

In this study we used simulated microgravity conditions created by the RPM for 3D tissue engineering of MCS and investigated the underlying mechanisms for a scaffold-free 3D growth behaviour of human breast cancer cells. Under normal laboratory conditions single cancer cells grow as monolayers. If sophisticated methods of tissue engineering are applied, MCS can be obtained which resemble the original, individual tumor more closely than corresponding cell monolayers^22^. Most interesting are methods of tissue engineering, which do not need scaffolds exerting unfavorable side-effects^24^,^25^. A suitable method to trigger the formation of MCS is culturing cells under microgravity conditions which is best provided by space missions^9^,^26^. Space research such as human space exploration and research applying simulated microgravity using ground-based facilities have increased our knowledge in cellular and molecular biology and given us new insights into the behavior of human cells under altered gravity conditions^13^,^27^,^28^. Devices simulating microgravity allow performing rather effective scaffold-free tissue engineering experiments in a much cheaper way and with a higher throughput^13^,^22^. It has been observed that normal and human thyroid cells grew in form of an adherent monolayer and as 3D aggregates^7^. This observation is in concert with results obtained by others as well as by our group. For example, when cultured on the RPM endothelial cells, murine osteoblasts and human breast cancer cells split into two populations with different phenotypes, respectively^18^,^29^,^30^. To explain this special behavior the non-equilibrium thermodynamics theory will be discussed. The non-equilibrium thermodynamics or birfurcation theory is describing the direct action of gravity on single cells^31^,^32^.

Biochemical reactions catalyzed by enzymes and controlled by feedback mechanisms in the organism are nonlinear and far away from an equilibrium. Therefore, a cell may answer unexpectedly to changed conditions of the cellular microenvironment. The key elements of a reaction remain constant and they can react with a known phase, frequency or amplitude. This crossroad or bifurcation system is depending on the microenvironment^31^,^32^. Microgravity can influence the cell to react in a different way compared with cells cultured under static normal 1 *g*-conditions. A large number of publications of studies performed in simulated and real microgravity in space have shown that different kinds of cells exhibit dramatic changes after microgravity-exposure^22^,^33^.

Since a long time scientists observed changes in a variety of cellular biological processes, such as apoptosis or angiogenesis. The cells showed changes in cell morphology, growth behavior, proliferation, differentiation, cell adhesion, extracellular matrix, among others^34^,^35^,^36^,^37^,^38^,^39^.

Furthermore, it is known that microgravity induces alterations in the cytoskeleton^34^,^38^,^39^. These cytoskeletal changes occur early as shown by parabolic flight maneuvers^40^,^41^. In addition, alterations in the actin cytoskeleton have been detected in space-flown xenopus embryonic muscle cells which exhibited marked changes in the distribution and organization of actin filaments^42^. These alterations of the actin cytoskeleton and microtubules are accompanied by changes of the shape of the cells^42^,^43^,^44^. This could already be seen after a 2 h-RPM exposure (Fig. 3). F-actin staining showed that AD cells on the RPM exhibited a membrane blebbing and at later time points stress fibers. After 24 h and 5d AD cells exhibited holes in the actin cytoskeleton. The cytoskeleton is sensing changes in gravity, and thus it is influencing signalling pathways^44^ and gene expression as well as protein synthesis and secretion^26^. Therefore, a variety of signaling processes such as cellular metabolism, proliferation, differentiation are changed when cells are cultured under altered gravity conditions^22^,^40^,^45^.

Real and simulated microgravity can directly or indirectly influence a cell^27^,^46^. Human cells are able to react to environmental changes. When, for example, cells are cultured on agarose in 96-well plates normal adherent cells grow in form of multicellular spheroids^47^ Under hypoxia or irradiation the cells will become apoptotic^48^. Altered gravity conditions have shown to influence gene expression, protein synthesis and the release of proteins in the cell supernatant in space^26^.

Changes in shape, cytoskeleton disruption, differential gene expression or altered protein synthesis/secretion cannot be only explained by considering changes in microenvironmental biophysical parameters. Gravitation might influence some general properties of the cells and thus acting “directly” as an organizing field parameter^46^. According to the non-equilibrium theory^49^ murine osteoblasts and breast cancer cells underwent a transition after a bifurcation point to new phenotypic configurations^18^,^30^. It is known for several cell types such as thyroid cancer cells, endothelial cells or chondrocytes that real and simulated microgravity induces two forms of growth, such as adherently growing cells and cells growing in form of 3D spheroids^6^,^8^,^13^,^29^,^50^. It has been demonstrated that some of the endothelial cells grown on the RPM form tubular intima-like structures^24^,^51^. In this paper the MCF-7 cells grew adherently after RPM-exposure and also in form of 3D aggregates after 24 h and showed gland-like structures after 5d as demonstrated in Fig. 2 by histochemical staining. The cells show an apical-basal polarity. To establish cell polarity, the MCF-7 cells have to interact with the surrounding medium, their neighbor cells and the ECM. Two main events are necessary for the development of 3D glandular structures. First the communication of the cells to the ECM and second the formation of a lumen. This might be possible with the interaction of integrins with laminin, which is like collagen type IV a constituent of the basement membrane. Both are known to efficiently induce the polarization of epithelial cells^52^,^53^,^54^ We measured an increase in *LAMA3* gene expression after 24 h in MCS (Fig. 8A) and after 5d an up-regulation of *LAMA3* in MCS compared with AD. This finding indicates that laminin may be involved in producing apical-basal polarity and the development of glandular structures. Collagen type IV was not significantly changed during all selected time points. Future investigations are necessary to study this process in more detail.

In this study, a few compact small aggregates had been detected in 24 h-RPM-cultures. This process starts after 16 h of RPM-exposure. MCF-7 cells appear to be very sensitive to RPM-exposure. A 24 h-period of microgravity is sufficient to induce a multitude of adaptive mechanisms inside the cells^6^. Similar to breast cancer cells (Fig. 1), two cell populations of FTC-133 thyroid cancer cells could be detected in RPM cultures: 2D growing adherent cells and floating 3D spheroids. Both populations exhibited a different growth behavior and signaling. Most interestingly, the adherent cells showed the highest rate of apoptosis and the most prominent gene expression of NF-kB, while the genomic profile of MCS cells appeared closer to that of 1 *g*-control cells than AD cells^6^. Similar results were found for endothelial cells^24^.

A microgravity-dependent inhibition of cancer cell proliferation, migration, and survival was found in MCF-7 cells and poorly differentiated follicular thyroid cancer cells^15^,^16^,^39^,^55^. This effect is cell type-dependent. For example chondrocytes reveal a decrease in apoptosis after simulated microgravity-exposure as well as fetal fibroblasts^56^,^57^. As already mentioned, MDA-MB-231 breast cancer cells exposed to the RPM also revealed two distinct phenotypes after 24 h^18^. A similar finding was observed in osteoblasts and chondrocytes cultured in microgravity^30^,^58^. This could be confirmed for MCF-7 breast cancer cells in this study. Moreover, we detected glandular structures which were only detected in long-term cultures. The first phenotype remained adherent to the cell culture flask as shown in Fig. 1. These cells exhibited a similar morphology as the 1 *g*-control cells. The second phenotype had detached from the bottom and was growing in form of 3D spheroids floating in the supernatant as earlier shown for thyroid cancer cells after RPM- or clinostat-exposure^6^,^7^,^10^,^47^. In this study, we investigated the F-actin cytoskeleton and the expression of genes coding for proteins which might be involved in the perception of gravity and the formation of MCS^6^,^7^,^8^,^9^,^10^,^24^. Between 16 h and 24 h MCF-7 cells started to form small round 3D aggregates. We could show for the first time that a part of the MCF-7 cells exhibited duct-like MCS after 5d on the RPM. These structures seemed to resemble a gland-like appearance to be found in mammary glands known as *alveoli* which align in lobules^59^. Before, we had repeatedly observed that cell populations split on the RPM into one subpopulation staying adherent to the bottom of the culture dish and another population, which had detached from the bottom and built up MCS (Figs 1, 2, 3)^7^,^50^. However, only healthy cells like the chondrocytes or the endothelial cells formed structures resembling the original cartilage tissue or the blood vessel intima, respectively^8^,^51^, while culturing de-differentiated human thyroid cancer cells always led to rounded spheres^5^,^7^,^9^. The gland-like appearance of MCS formed by MCF-7 cells might be due to a high differentiation status of the MCF-7 cells, which still express estrogen and progesterone receptors^17^,^21^. Moreover, other studies suggested that simulated microgravity enacts the reversion of the neoplastic phenotype of lung cancer stem cells^60^. This is supported by studies investigating cancer cells in a different tissue environment. This model of tumor reversion has demonstrated a clinical benefit in hematological malignancies. These cells show a shift from a neopalstic toward a normal phenotype^61^. Cell morphology of cells growing as monolayer in 1 *g*-cultures is different from those observed in tissue and organs. The shape of the cells is influenceing cell growth, cell metabolism as well as gene expression and thus, this might explain the differences in the gene expression of AD and MCS cells^62^.

In addition, it was described that either induced by drugs or by fibroblasts, MCF-7 may adopt almost normal biochemical characteristics and form nodules^63^,^64^. Moreover, a possible microgravity-dependent change in the cellular differentiation of the cells was suggested by the results obtained after dedifferentiated thyroid cancer had been flown on the Shenzhou-8 spaceflight^26^.

Although the expression of estrogen receptors is a marker of the high-differentiation status of the MCF-7 cells, their presence may promote breast cancer proliferation under some circumstances^65^. However, the simultaneous presence of the progesteron receptor often attenuates the proliferative action of the estrogen receptor switching a tumor cell to a more differentiated state^66^. We found an up-regulation of the *ESR1* gene expression in AD cells after a 5d-RPM-exposure. The *ESR1* gene remained unchanged during the short-term study. The expression of *PGR1* was up-regulated in both phenotypes of 5d-RPM-samples (Fig. 9). The up-regulation of both hormone receptors is transient like the production of caspase-3 in thyroid cells^67^. An upregulation of *PGR1* genes in MCF-7 MCS and AD cells seem not to influence the fibronectin promoter like in fibroblasts^68^. *FN1* was down-regulated after 5d in AD and MCS, which could be a cause that also kinase insert domain receptor (*KDR*) gene is down-regulated in AD and MCS, because its gene status is under the positive influence of *FN1* as shown in Fig. 4 and described in the literature^69^,^70^,^71^,^72^,^73^.

Interestingly, according to the Elsevier Pathway Studio analysis *FN1, KDR,* and *ICAM1* genes code for proteins which are members of a signalling pathway that regulates the shape of the cells, while MSN-RDX-proteins and ICAM1-MSN-proteins together with EZR, respectively, participate in triggering tip formation of cells and membrane to membrane docking. Cell shape changes and renewed membrane to membrane docking can clearly be seen in Figs 1, 2 and 3.

It is known that cancer cells actively remodel their ECM and that the cell adhesion molecule fibronectin is important for survival signaling, progression and metastasis in breast cancer cells^74^. Thyroid cancer cells (FTC-133 cell line) express surface proteins that bind fibronectin, strengthening the 3D cell cohesion^5^. The moesin gene is regulated like *FN1* (Figs 6F and 7B). This parallel regulation may be due to miR-200c, which can target *FN1* as well as *MSN*^75^. Ezrin, radixin and moesin belong to the ERM protein family and connect the plasma membrane with the actin cytoskeleton and are therefore of high interest to be part of the external signal transport into the cell^76^. The ERM proteins are strongly associated with ICAM1^77^,^78^. A suppression of ERM proteins resulted in a destruction of cell-cell and cell-substrate adhesion, while an overexpression enhanced cell adhesion^78^. Hence, the decreased expression of ERM genes after a 5d-exposure might be a reason for the detachment of the cells and the undefined appearance of the MCS.

This transition process is accompanied by a rearrangement of the actin (Fig. 3) as it has been shown earlier in spaceflight-MCF-7-samples^14^. In this study, the 1 *g*-control cells presented visible filaments, like it was seen in endothelial cells and thyroid cells. In duct-like MCS the actin filament systems of the MCF-7 cells is more similar to actin filament systems of tubes formed by EAhy cells than to that formed by thyroid cancer cells^29^,^47^. Actin as well as tubulin and keratin are major parts in the cytoskeleton bearing different functions and are thought to be critical for the perception and forwarding of external chemical and physical signals like the gravity force^44^.

Gravity is known to influence directly or indirectly the behavior of cells^46^. It follows nonequilibrium dynamic rules^49^. A nonequilibrium reaction influenced by gravity in living cells is represented by the dynamices of the cytoskeleton, by shape and differentiation of the cells^30^. Epithelial cell migration is regulated by three major signaling nodes, β-catenin, integrin-β_1_ and actin^79^. Many of these genes are involved in cell-cell and cell-matrix adhesion through regulation of the actin cytoskeleton and EGFR signaling^79^.

MCF-7 cells exposed to the RPM for 2 h, 4 h, 16 h and 24 h exhibited no changes in the gene expression of cytoskeletal genes. In contrast, we found β-actin and β-tubulin genes in MCS to be significantly down-regulated after 5d. Simultaneously, the amount of β-actin protein was elevated in AD and MCS cells after 5d, while equal amounts of tubulin were found in all measurements. In addition, *KRT8*, which is a luminar marker^80^, was equally expressed after 5d of cultivation on the RPM as in 1 *g*-controls. Densitomertric analyses revealed a higher content of keratin 8 (subunit 46 kDa) in AD and MCS after 5d compared to their corresponding 1 *g*-controls. Moreover, up-regulation in β-actin gene expression had also been found for human thyroid cells cultured on the RPM for 7 and 14 days^7^. However, a direct mutual influence of these three cytoskeletal proteins could not be seen on the gene nor on the protein level.

Integrins are known to crosslink with ICAM1 and co-localizes with moesin in microvilli in endothelial cells^81^. By this, the small GTPase RhoA is activated which induces the production of stress fibers and the up-regulation of the *rhoA* gene^82^. We found *ITGB1* up-regulated in 4 h-AD-samples. Later *ITGB1* was significantly down-regulated in 24 h-MCS and then non-significantly down-regulated after 5d in RPM-samples as in 1 *g*-samples. The densitomertric analyses presented a slight decrease in integrin-β_1_ protein after 5 days. Integrin- β_1_ is a membrane protein, linking to the extracellular matrix with the cytoskeleton and is capable of transmitting signals^83^. Primarily found in focal adhesions it is described to be pivotal to activate signalling pathways which lead to differentiation, angiogenesis, proliferation and cytoskeleton rearrangements among others^81^. Our findings suggest that integrin-β_1_ is involved in the detachment of the cells from the culture flask. The ECM is also involved in tissue polarity and architecture. Integrin-β_1_ maintains this polarity in the mammary gland^84^. The integrins’ extracellular interaction with the ECM and intracellular interactions with the cellular cytoskeleton, are examples of cellular mechano-transducers^85^.

It has been shown that an abnormal ECM promotes the formation of a tumor microenvironment and plays a role in tumor angiogenesis. ECM components are involved in vessel formation and ECM fragments deriving from collagen type IV and others can influence angiogenesis. They interact with angiogenesis signaling factors including VEGF to initiate vascular branching^84^.

In this study, the expression and release of VEGF after a 4 h-, 24 h- and 5d-RPM-exposure was significantly reduced compared to the corresponding controls. This is in concert to previous experiments using thyroid cancer cells which presented a decrease in VEGF, however, experiments using endothelial cells presented an upregulation of VEGF^10^,^26^,^50^,^86^. Its signaling pathways control survival, proliferation, and migration and actin reorganization among others^87^. Their interruptions may have beneficial effects to cancer patients^88^. VEGFR1 expression was not altered, while VEGFR2 presented a down-regulation in both 5-day-populations. This suggests that the VEGFR2 is the more important VEGF receptor, when MCF-7 cells react to microgravity. Binding of VEGF to VEGFR activates MAPK and Akt1 pathway which are responsible to control proliferation and survival^89^. *Akt1* gene expression was not altered after a 5-day-exposure, however, the downstream signalling molecules caspase-9 and caspase-3 were significantly down-regulated which can hint to a higher survival rate as less protein was produced. This finding indicates a higher survival rate of adherent cells compared to the cells accumulated to MCS. The MAPK/ERK pathway inherits PRKCA and downstream ERK1/ERK2 controlling proliferation among others. *PRKCA* as well as *ERK1* were down-regulated after 5 days on the RPM. Taken together, the VEGF-dependent pathways do not seem to be the dominant driving force, when MCF-7 cells transit from the 2D to 3D kind of growth.

Instead of VEGFA, IL-8 could be a potential key-player, as it is capable of acting on a variety of different cell types while being elevated in some tumour types^47^ (Fig. 4). IL-8 expression is associated with a higher invasiveness potential of breast cancer cells *in vitro*, proposing IL-8 as a novel marker of tumor aggressiveness^90^.

IL-8 is involved in 3D-aggregate-formation in thyroid cancer cells^47^. In this study, we found the *IL8* gene expression to be elevated after 2 h and 4 h in RPM-exposed adherent cells, then after 24 h up-regulated in MCS, but later unaltered in AD and slightly decreased in MCS after a 5d-exposure. IL-8 has recently been shown to modulate breast cancer invasion and angiogenesis^91^. Further studies will be performed to increase our knowledge of its role in 3D formation.

Also the *RhoA* gene expression does not seem to be involved in the alteration of growth of MCF-7 cells, because its gene was unaltered as compared to the 1 *g*-controls after a 5-RPM-exposure. Further experiments are required to descramble the network of regulation of genes and proteins triggering MCS formation in MCF-7 cells.

## Conclusion

The exposure of human cells to simulated microgravity conditions created by an RPM has an enormous influence on their morphology and biology. It seems, that upon cultivation on the RPM the cells transform from monolayer into their *in vivo* typical 3D tissues. The formation of multicellular spheroids of MCF-7 breast cancer cells after RPM-exposure starting between 16 and 24 h is an important finding. After a 5d-RPM-exposure glandular structures are visible. In this process, disconnecting and reconnecting cell-cell connections as well as a strong regulation of the cell shape appear important. It has to be clarified in the future, whether gravity influences human cancer cells directly or indirectly. The non-equilibrium theory may explain how the cytoskeleton is sensitive enough to sense gravity changes, and induces the transfer of the mechano-signal into biochemical pathways^46^. Recently, it was shown that during a rocket flight and a parabolic flight live cell imaging of LifeAct-GFP-transfected thyroid cancer cells revealed significant alterations of the cytoskeleton related to microgravity^92^. Life-cell imaging during microgravity proved early changes in the actin cytoskeleton in real microgravity, which was described earlier on fixed cells.

Future investigations, using additional growth factors for histological and functional investigations, will show, if cultivation of normal breast cells on the RPM can produce functionally active breast tissues. Furthermore, impending studies should further investigate the possiblity of using fibronectin and IL8 as novel future targets in the treatment of breast cancer.

## Methods

### Cell culture

MCF-7 human breast adenocarcinoma cells were purchased from the American Type Culture Collection (MCF7 (ATCC^®^ HTB-22^™^)) and cultivated in RPMI 1640 (Life Technologies, Naerum, Denmark) medium supplemented with 10% fetal calf serum (FCS) (Biochrom AG, Berlin, Germany) and 1% penicillin/streptomycin (Life Technologies) and maintained under standard cell culture conditions at 37 °C and 5% CO_2_. One day prior to the RPM experiment, 1 × 10^6^ cells were counted and seeded into T25 cm^2^ vented cell culture flasks (Sarstedt, Newton, USA) or 2.5 × 10^5^ cells were seeded into slide flasks (Thermo Scientific, Roskilde, Denmark) for F-actin cytoskeleton investigations. Each flask was completely filled with medium, taking care that no air bubbles remained in the cell culture flasks. The flasks were installed on the centre plate of the RPM and run for 2 h (n = 30), 4 h (n = 30), 16 h (n = 30), 24 h (n = 30) and 5d (n = 30) respectively using the real random mode. 1 *g-*static controls were prepared in parallel (n = 30 each group) and stored next to the device in the same incubator.

After each time point the cells were investigated by phase contrast microscopy and photographed. The supernatant was collected and centrifuged at 4 °C to collect the MCS. After centrifugation the supernatant was collected for cytokine investigation on ice and then frozen at −20 °C. The MCS were collected and stored in liquid nitrogen.

For harvesting the adherent cells, 5 ml of ice-cold phosphate buffered saline (PBS) (Life Technologies) was carefully added to each T25 cm^2^ flask and the cells were scraped off with a scraper. The cell suspension was collected and centrifuged at 4 °C. The PBS was discarded and the dry pellet was stored in liquid nitrogen.

### Random Positioning Machine (RPM)

The RPM (ADS, former Dutch Space, Leiden, Netherlands) was run in a commercially available incubator at 37 °C and 5% CO_2_. The method was intensively investigated and published earlier^5^,^6^,^10^,^26^. Mode of choice was the real random mode with random speed and random interval and a maximum speed of 75°/s. 15 T25 cm^2^ flasks were fixed to the operating platform, resulting in a maximal distance of 7.5 cm to the rotation axis, and were rotated for the selected time periods respectivly. Static, non-rotated controls were exposed to the same environmental conditions nearby the device.

### Phase contrast microscopy

Phase contrast microscopy was performed for visual observation of the morphology of the cells, using a Leica (Microsystems GmbH, Wetzlar, Germany). Pictures had been taken by a Canon EOS 550D (Canon GmbH, Krefeld, Germany).

### F-actin cytoskeleton staining

Cells exposed for 2 h, 4 h, 16 h, 24 h and 5d to the RPM in slideflasks were investigated. F-actin was visualized by means of rhodamine-phalloidin staining (Molecular Probes®, Eugene, OR, USA). In addition, the nuclei were stained with 4′,6-diamidino-2-phenylindole (DAPI, Life Technologies). The method was described earlier in detail^47^,^93^.

### Hematoxylin-Eosin and Periodic acid–Schiff staining

After a 5d-RPM-culture of MCF-7 cells, the MCS were collected, three times carefully washed in PBS and fixed in 4% paraformaldehyde. The MCS were embedded in paraffin^94^ and sectioned with a microtome. The MCS were cut into 3 μm sections. Hematoxylin and eosin stains were used to evaluate the cell morphology and polarity of the breast cancer cells. In addition, the Periodic acid-Schiff (PAS) was applied to investigate the cellular basement membranes of the MCS cells. All sections were visualized by light microscopy using an oil immersion objective with a calibrated magnification of ×400.

### NGAL and VEGF measurements

NGAL and VEGF levels were measured using in-house time resolved immunofluorometric assays (TRIFMA) according to previously described methods^7^,^95^. For all determinations, the supernatant samples were diluted 1:2 and the 96-well plates were read using a VICTOR 2030 (Perkin Elmer, Inc.) Standard curves were used to calculate the concentrations using the standard software implemented in the VICTOR 2030.

### RNA and protein isolation and quantitative real-time PCR

RNA isolation and quantitative real-time PCR were performed according to routine protocols^7^,^25^,^94^. RNA and protein were isolated using the AllPrep RNA/Protein kit (Qiagen GmbH, Hilden, Germany) following the manufacturer’s instructions. The RNA was quantified via the SpectraMax M2 (Molecular Devices, California, USA). Reverse transcription was performed using the First Strand cDNA Synthesis Kit (Thermo Scientific, Waltham, Massachusetts, USA) following manufacturer’s instructions. Quantitative real-time PCR was utilized to determine the expression levels of target genes, shown in Table 1, using the SYBR® Select Master Mix (Applied Biosystems, Darmstadt, Germany) and the 7500 Real-Time PCR System (Applied Biosystems). cDNA-selective Primers were designed to span exon-exon boundaries and to have a Tm of 60 °C using Primer Express software (Applied Biosystems), and were synthesized by TIB Molbiol (Berlin, Germany). All samples were measured in triplicate and normalized to the housekeeper 18S rRNA. Comparative CT (ΔΔCT) methods were used for relative quantification of transcription levels, with 1 *g* set as 100%.

### Western blot analysis

Gel electrophoresis, trans-blotting, and densitometry were carried out following routine protocols as described previously^94^. 20 μL of lysate containing 2 μg/μL protein was loaded onto SDS-PAGE per sample. A total number of 5 samples were analyzed per cell population for 5 and 10 days respectively. Primary antibodies were applied as described in Table 2. HRP-linked, secondary antibody was used at a dilution of 1:3000 (Cell Signaling Technology, Inc., Danvers, MA, USA). A final analysis was performed in a ChemiDoc XRS+ (Bio Rad, Hercules, CA, USA). To quantify the bands desitometrically, the membranes were analysed using ImageJ software (U.S. National Institutes of Health, Bethesda, MD, USA).

### Pathway Studio Analysis

Pathway Studio v11 was purchased from Elsevier Research Solutions, Amsterdam, Netherlands. This program was used online^96^. To start an analysis, the SwissProt numbers of the proteins of interest were entered^97^.

### Statistical Evaluation

All statistical analyses were performed using SPSS 21.0 (SPSS, Inc., Chicago, IL, USA, 2012). The data was analyzed with the Mann-Whitney U test. To account for multiple comparisons, a Kruskal-Wallis Test was performed beforehand, and Bonferroni corrections were applied. The data was expressed as means ± standard deviation (SD). Differences were considered significant at p < 0.05.

## Additional Information

**How to cite this article**: Kopp, S. *et al*. Identifications of novel mechanisms in breast cancer cells involving duct-like multicellular spheroid formation after exposure to the Random Positioning Machine. *Sci. Rep.* **6**, 26887; doi: 10.1038/srep26887 (2016).

## Figures and Tables

**Table 1:** Primers used for qRT-PCR.

**Gene**	**Primer Name**	**Sequence**
*18S rRNA*	18S-F	GGAGCCTGCGGCTTAATTT
	18S-R	CAACTAAGAACGGCCATGCA
*ACTB*	ACTB-F	TGCCGACAGGATGCAGAAG
	ACTB-R	GCCGATCCACACGGAGTACT
*Casp3*	Casp3-F	AACTGCTCCTTTTGCTGTGATCT
	Casp3-R	GCAGCAAACCTCAGGGAAAC
*Casp9*	Casp9-F	CTCCAACATCGACTGTGAGAAGTT
	Casp9-R	GCGCCAGCTCCAGCAA
*CD44*	hCD44-F	ACCCTCCCCTCATTCACCAT
	hCD44-R	GTTGTACTACTAGGAGTTGCCTGGATT
*Col4A5*	Col4A5-F	GGTACCTGTAACTACTATGCCAACTCCTA
	Col4A5-R	CGGCTAATTCGTGTCCTCAAG
*ERK1*	ERK1-F	ACCTGCGACCTTAAGATTTGTGA
	ERK1-R	AGCCACATACTCCGTCAGGAA
*ERK2*	ERK2-F	TTCCAACCTGCTGCTCAACA
	ERK2-R	TCTGTCAGGAACCCTGTGTGAT
*ESR*	ESR1-F	TTCAAGAGAAGTATTCAAGGACATAACG
	ESR1-R	TCGTATCCCACCTTTCATCATTC
*EZR*	EZR-F	GCAATCCAGCCAAATACAACTG
	EZR-R	CCACATAGTGGAGGCCAAAGTAC
*FLK1*	hFLK1-F	TCTTCTGGCTACTTCTTGTCATCATC
	hFLK1-R	GATGGACAAGTAGCCTGTCTTCAGT
*FLT1*	FLT1-F	CCCTCGCCGGAAGTTGTAT
	FLT1-R	GATAATTAACGAGTAGCCACGAGTCAA
*FN1*	FN-F	AGATCTACCTGTACACCTTGAATGACA
	FN-R	CATGATACCAGCAAGGAATTGG
*ICAM1*	ICAM1-F	CGGCTGACGTGTGCAGTAAT
	ICAM1-R	CTTCTGAGACCTCTGGCTTCGT
*IL8*	IL8-F	TGGCAGCCTTCCTGATTTCT
	IL8-R	GGGTGGAAAGGTTTGGAGTATG
*ITGB1*	ITGB1-F	GAAAACAGCGCATATCTGGAAATT
	ITGB1-R	CAGCCAATCAGTGATCCACAA
*KRT8*	KRT8-F	GATCTCTGAGATGAACCGGAACA
	KRT8-R	GCTCGGCATCTGCAATGG
*LAMA3*	LAMA3-F	AAAGCAAGAAGTCAGTCCAGC
	LAMA3-R	TCCCATGAAGACCATCTCGG
*MSN*	MSN-F	GAAATTTGTCATCAAGCCCATTG
	MSN-R	CCATGCACAAGGCCAAGAT
*NGAL*	NGAL-F	AGGGAGTACTTCAAGATCACCCTCTA
	NGAL-R	AGAGATTTGGAGAAGCGGATGA
*PARD3*	PARD3-F	TACAGTGGGATTGAGGGGCT
	PARD3-R	GCTGGTATTTACCTGACTCACC
*PARD6A*	PARD6A-F	ATACGGATGCTCATGGCGAC
	PARD6A-R	GTCAGCTTCTGCCCGCTTCT
*PKB*	AKT1-F	CTTCTATGGCGCTGAGATTGTG
	AKT1-R	CAGCATGAGGTTCTCCAGCTT
*PKC*	PKC-F	CATTCAACAGCTGGGCAAGTT
	PKC-R	GTAGATGATGCCCTGATTGTGAAG
*PGR*	PGR-F	GTGGGAGCTGTAAGGTCTTCTTTAAGA
	PGR-R	TGACAGCACTTTCTAAGGCGACA
*PRKCI*	PRKCI-F	GTGTAAGGAAGGATTACGGCCA
	PRKCI-R	GCCCACCAGTCAACACTGAA
*RDX*	RDX-F	GAAAATGCCGAAACCAATCAA
	RDX-R	GTATTGGGCTGAATGGCAAATT
*RhoA*	RhoA-F	CGTTAGTCCACGGTCTGGTC
	RhoA-R	GCCATTGCTCAGGCAACGAA
*TUBB*	TUBB-F	CTGGACCGCATCTCTGTGTACTAC
	TUBB-R	GACCTGAGCGAACAGAGTCCAT
*VEGFA*	VEGFA-F	GCGCTGATAGACATCCATGAAC
	VEGFA-R	CTACCTCCACCATGCCAAGTG

**Table 2:** Primary antibodies used for Western blot analyses.

**Protein**	**Dilution**	**Company**
*β-actin*	1:1000	Cell Signaling Technology, Inc., Danvers, MA, USA
*β-tubulin*	1:100	Sigma Aldrich, St. Louis, MO, USA
*Ezrin*	1:1000	Cell Signaling Technology, Inc., Danvers, MA, USA
*Fibronectin*	1:1000	Sigma Aldrich, St. Louis, MO, USA
*GAPDH*	1:1000	Cell Signaling Technology, Inc., Danvers, MA, USA
*Integrin-b*_*1*_	1:1000	Cell Signaling Technology, Inc., Danvers, MA, USA
*Keratin*	1:1000	Cell Signaling Technology, Inc., Danvers, MA, USA
*Laminin*	1:1000	Sigma Aldrich, St. Louis, MO, USA
*Radixin*	1:1000	Cell Signaling Technology, Inc., Danvers, MA, USA
*VEGF*	1:200	Abcam plc, Cambridge, UK
